# SK-216, a Novel Inhibitor of Plasminogen Activator Inhibitor-1, Suppresses Lung Metastasis of Human Osteosarcoma

**DOI:** 10.3390/ijms19030736

**Published:** 2018-03-05

**Authors:** Minori Tsuge, Mitsuhiko Osaki, Ryo Sasaki, Mio Hirahata, Futoshi Okada

**Affiliations:** 1 Division of Pathological Biochemistry, Department of Biomedical Sciences, Faculty of Medicine, Tottori University, 86 Nishi-cho, Yonago, Tottori 683-8503, Japan; ts.mino.71@gmail.com (M.T.); ry-x.y.z.117@hotmail.co.jp (R.S.); mio_cute_animals@hotmail.com (M.H.); fuokada@med.tottori-u.ac.jp (F.O.); 2 Chromosome Engineering Research Center, Tottori University, 86 Nishi-cho, Yonago, Tottori 683-8503, Japan

**Keywords:** osteosarcoma, metastasis, PAI-1, small molecule inhibitor

## Abstract

Lung metastasis constitutes the leading cause of the death in patients with osteosarcoma. We have previously reported that plasminogen activator inhibitor-1 (PAI-1) regulates the invasion and lung metastasis of osteosarcoma cells in a mouse model and as well as in clinical samples. In the present study, we examined the anti-metastatic effect of SK-216, a small compound PAI-1 inhibitor, in human 143B osteosarcoma cells. An in vitro study showed that SK-216 treatment suppressed invasion activity by inhibiting PAI-1 expression in 143B cells, but had no influence on their proliferation or migration. 143B cells treated with SK-216 exhibited reduced matrix metalloproteinase-13 (MMP-13) secretion in a dose-dependent manner. Moreover, intraperitoneal injection of SK-216 into mouse models resulted in downregulation of PAI-1 expression levels in the primary tumors and showed suppression of lung metastases without influencing the proliferative activity of the tumor cells in the primary lesions. These results indicate that SK-216, a PAI-1 inhibitor, may serve as a novel drug to prevent lung metastasis in human osteosarcoma.

## 1. Introduction

Osteosarcoma is the most common primary bone tumor that primarily affects children and adolescents. Although the prognosis of patients with osteosarcoma has improved through the use of combination therapy such as adjuvant chemotherapy in conjunction with surgical wide resection [1], reported five-year survival rates for patients with lung metastasis remain at approximately 30% [2]. As lung metastases constitute the leading cause of death in patients with osteosarcoma, it is therefore important to suppress lung metastases in osteosarcoma for improving their prognosis.

Toward this end, our group previously reported that intravenous injection of miR-143 significantly suppressed the lung metastasis of human 143B osteosarcoma cells in a mouse model [3]. Moreover, we identified plasminogen activator inhibitor-1 (PAI-1) as a direct target gene of miR-143 [4]. PAI-1 is a serine protease inhibitor with a main role as a regulator of tissue-type and urokinase-type plasminogen activator proteolytic activity during coagulation [5]. In addition, high levels of PAI-1 are widely reported to be positively correlated with poor clinical outcome in various cancers [6]. Our prior findings also indicated that PAI-1 regulates the invasion and lung metastasis of 143B cells in a mouse model [4], suggesting that PAI-1 might serve as a target gene for preventing lung metastasis of osteosarcoma.

It has been reported that the small compound PAI-1 specific inhibitor, SK-216, could suppress PAI-1 expression in rat colon cancer cells as well as intestinal polyp formation in a Min mouse [7]. Furthermore, SK-216 could inhibit lung metastasis of human lung cancer cells and mouse melanoma cells in an intravenously-injected mouse model [8]. However, no studies have been reported regarding the potency of SK-216 against the lung metastasis of human osteosarcoma cells. Accordingly, the aim of the present study was to evaluate the suppressive effect of SK-216 on the invasion and lung metastasis of human osteosarcoma cells.

## 2. Results

### 2.1. SK-216 Inhibits the Invasion of 143B Cells In Vitro

**Figure 1:**
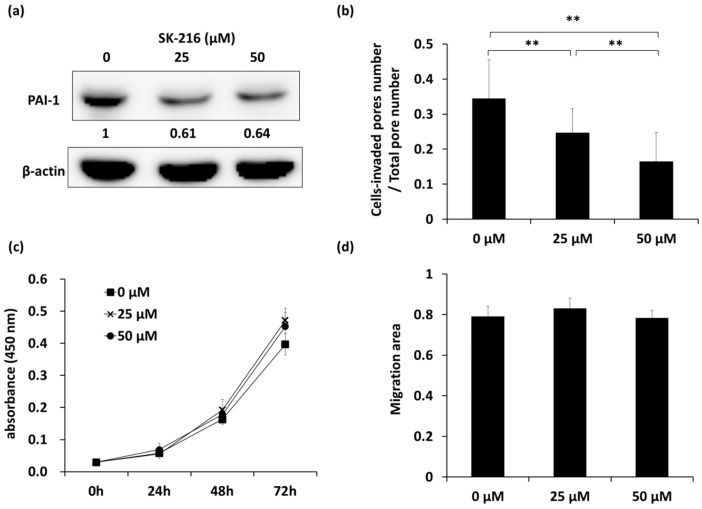
The PAI-1 inhibitor SK-216 suppresses invasion with no influence on proliferation or migration of 143B cells. (**a**) Western blot analyses of PAI-1 expression in SK-216 treated 143B cells. PAI-1 expression was quantified using Image Studio Lite (LI-COR) and normalized to β-actin. Expression is shown relative to that in non-treated cells (0 μM); (**b**) Matrigel assay of the invasion of SK-216-treated cells. The ratio of the number of pores containing invading cells to the total number of all pores is shown. Bar graphs show means ± SD ** *p* < 0.01; (**c**) Proliferation assay indicating absorbance (450 nm) measured at 0, 24, 48, or 72 h after SK-216 treatment for 143B cells is shown (*n* = 5 wells per group); (**d**) Scratch assay of the migration of 143B cells after 48 h treatment of SK-216. The migrated areas were analyzed at about 30 h after being scratched. Bar graphs show means ± SD.

We first examined the inhibitory effects of SK-216 on the invasion, proliferation, and migration ability of human osteosarcoma cells. Western blotting showed that there was approximately 40% inhibition of PAI-1 expression in 143B cells that were treated with SK-216 at 25 and 50 μM when compared with non-treated cells (Figure 1a, Supplementary Figure S1a). A Boyden Chamber assay with Matrigel coating revealed a significant decrease in the ratio of cell-invaded pores/total pores following SK-216 treatment (from 0.34 in non-treated cells to 0.25 and 0.16 in 25 and 50 μM SK-216 treated cells, respectively), indicating that SK-216 inhibited the invasion of 143B cells in a dose-dependent manner (Figure 1b, Supplementary Figure S1b, *p* < 0.01). Conversely, SK-216 affected neither cell proliferation nor migration (Figure 1c,d, Supplementary Figure S1d). Additionally, we also ascertained the similar effect of SK-216 on HOS, another osteosarcoma cell line (Supplementary Figure S1a,c). These data indicated that SK-216 could reduce PAI-1 expression and consequently suppress the invasion, but not the proliferation or migration, of human osteosarcoma cells.

### 2.2. SK-216 Suppresses Lung Metastasis of Osteosarcoma Cells In Vivo

**Figure 2:**
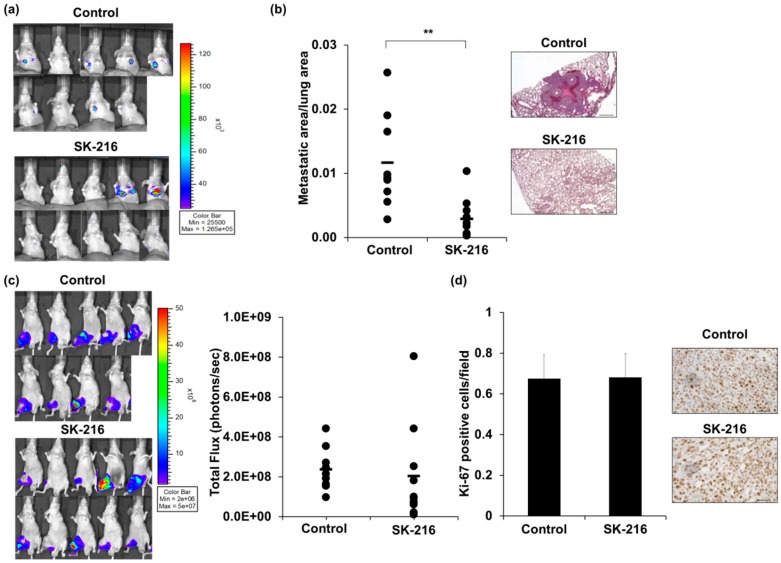
Intraperitoneal injection of SK-216 suppresses lung metastasis of 143B cells in a mouse model. (**a**) 143B-Luc cells were inoculated into the right knee of a model mouse. Lung metastases at 5 weeks after inoculation are reflected in bioluminescence; (**b**) the areas of metastatic lesion on the lung in the model mice were plotted. The black bar indicates the mean value. Representative hematoxylin and eosin (H & E) staining of the lung at five weeks after inoculation are shown. Scale bars, 500 μm; (**c**) Primary tumors at five weeks after cell-inoculation are reflected in bioluminescence. Total Flux (photons/seconds) measured in the obtained IVIS images of mice. The black bar indicates the mean value; (**d**) the rates of tumor cells that were positive for Ki-67 in primary tumors were calculated by counting 10 visual fields at high magnification. Representative staining of Ki-67 from control and SK-216 treated mice are shown. Scale bars, 50 μm.

Next, we examined whether SK-216 suppresses lung metastasis of human osteosarcoma cells using a spontaneous lung metastasis mouse model [3]. 143B-Luc cells were inoculated into the right knee and SK-216 (6.6 μg/200 μL) or PBS (phosphate buffered saline) as a control was administered intraperitoneally to each group once every three days. At five weeks after the cell inoculation, six of the nine mice (66.7%) in the control group displayed a luciferase signal at the pulmonary area, suggesting lung metastasis. In contrast, only two of the ten mice (20.0%) in the SK-216 treatment group displayed pulmonary signal (Figure 2a, Table 1), a significantly lower frequency than in the control group (*p* < 0.05), although there were no differences in the quantification of fluorescent signal at the pulmonary area between control and SK-216 treatment group (Supplementary Figure S2), it may owing to strong signals on two mice in SK-216 group. Moreover, macroscopic confirmation in the lung surface revealed that the number of metastatic lesions was significantly reduced in the SK-216 treatment group (Table 1, *p* < 0.05). In addition, histological analysis of lung metastasis revealed that the mean ratio of metastatic area to total lung area was significantly decreased in the SK-216 treatment group (approximately 0.3%) compared to the control group (1.2%, Figure 2b, *p* < 0.01). Conversely, there were no differences in cell proliferation between the control group and SK-216 treatment group, as determined by fluorescent signals and Ki-67 immunohistochemical staining of the primary tumor (Figure 2c,d). These data suggested that SK-216 could suppress lung metastasis of human osteosarcoma cells, but not tumor growth, in the primary lesion.

### 2.3. SK-216 Suppresses PAI-1 Expression of Osteosarcoma Cells In Vivo

**Figure 3:**
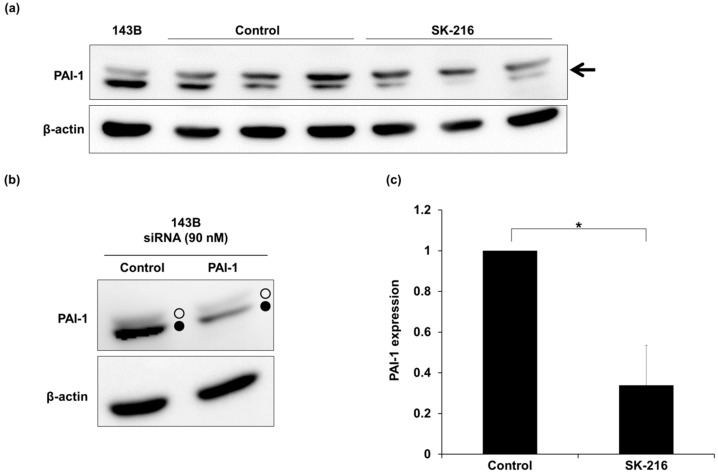
SK-216 suppresses PAI-1 expression of osteosarcoma cells in primary tumors. (**a**) Western blot analysis of PAI-1 expression (arrow) in primary tumors at 2 weeks after cell inoculation; (**b**) Western blot analysis of PAI-1 expression in 143B cells, which were transfected with PAI-1 siRNA or Control siRNA. Open circles and closed circles indicate upper and lower bands recognized by the anti-PAI-1 antibody used in this study, respectively; (**c**) PAI-1 expression in primary tumors was quantified using Image Studio Lite (LI-COR) and normalized to β-actin (±SD, *n* = 3 per group. * *p* < 0.05).

We then evaluated the effect of SK-216 on PAI-1 expression in primary tumors on day 14 after cell inoculation. Western blot analysis showed that two bands around 45 kDa were detected using an anti-PAI-1 antibody (Figure 3a). We confirmed that the lower band was a specific band according to a PAI-1-knockdown experiment (Figure 3b). Quantitative data showed that the PAI-1 expression level in primary lesions from SK-216-treated mice was significantly decreased when compared to that of control mice (Figure 3c, *p* < 0.05). These results showed that SK-216 administration could suppress lung metastasis of osteosarcoma cells by decreasing PAI-1 expression in the primary lesion.

### 2.4. SK-216 Suppresses MMP-13 Secretion

**Figure 4:**
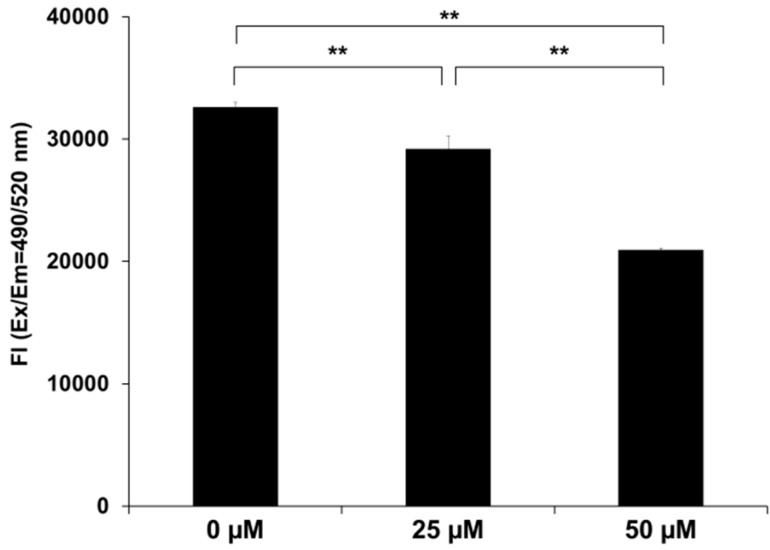
SK-216 attenuates MMP-13 secretion from 143B cells. MMP-13 levels in the conditioned media of SK-216-treated 143B cells were determined using the Sensolyte MMP-13 assay kit. The reaction was initiated by adding 50 μL of the substrate solution. The fluorescence intensity of the reaction (Fl) was determined by calculation of the ratio of λ emission (Em) = 485 nm/λ excitation (Ex) = 520 nm (±SD, *n* = 3 per group. ** *p* < 0.01).

Our previous report showed that PAI-1 regulates MMP-13 expression and secretion in 143B cells [4]. This finding motivated us to examine whether SK-216 might suppress MMP-13 secretion in 143B cells as well. As shown in Figure 4, we confirmed that the amount of MMP-13 secreted from 143B treated with SK-216 was also significantly lower in a dose-dependent manner than that secreted from non-treated cells (*p* < 0.01). These data indicated that the downregulation of PAI-1 by SK-216 resulted in a reduction of MMP-13 secretion in 143B cells.

## 3. Discussion

In the present study, we showed that SK-216, a PAI-1 inhibitor, suppressed the invasion and lung metastasis ability of human osteosarcoma cells through the reduction of MMP-13 secretion. MMP-13, a member of a zinc-dependent proteolytic enzyme family, has been associated with cancer progression and poor prognosis, such as head and neck [9], lung [10], and colorectal [11] cancers. In particular, MMP-13 contributes to bone lysis and metastasis in breast [12] and prostate [13] cancers. Osteolytic activity is necessary for osteosarcoma cells to expand tumor tissue from a primary lesion, followed by the promotion of invasion and metastasis. Notably, our data indicated that SK-216 suppressed cell invasion without influencing either cell proliferation or migration in osteosarcoma cells in vitro. In addition, the inhibitory effect of SK-216 on lung metastasis was not accompanied by a decrease in primary tumor volume in vivo. It was suggested that anti-proliferative effect via cell cycle arrest might not be induced by SK-216 in tumor cells as well as non-malignant cells (e.g., Human Umbilical Vein Endothelial Cells (HUVEC); a non-malignant human endothelial cell), as reported by Masuda et al. [8]. These results were equivalent to those that were obtained in our previous study using PAI-1 siRNA [4], suggesting that PAI-1 promotes cell invasion and metastasis and that PAI-1 represents a target molecule for preventing lung metastasis in human osteosarcoma.

SK-216 has been shown to suppress PAI-1 protein expression, suggesting that the PAI-1 inhibitory mechanism of SK-216 functions at the expression level as well as previous reports [7,14]. Thus, SK-216 mechanistically differs from most other PAI-1 inhibitors, which were designed to target one of the three PAI-1 protein-binding domains; reactive center loop, low density lipoprotein receptor-related protein 1-binding domain, and vitronectin-binding domain. For example, PAI-039 (tiplaxtinin), TM5275 and TM5441 comprise small molecule PAI-1 inhibitors targeting these domains that have been developed in the past decade and show anti-tumor effects. It has previously been reported that PAI-039 inactivated PAI-1 by disrupting its stable form by binding to its vitronectin-binding domain [15], inducing cell apoptosis in human lung cancer and fibrosarcoma in vitro [16]. It was also shown that PAI-039 suppressed tumor growth by promoting apoptosis and inhibiting angiogenesis in human bladder cancer and a cervical cancer xenograft model [17]. TM5275 has been reported to induce cell cycle arrest and apoptosis in human ovarian cancer cells in vitro [18], through the inhibition of interaction between PAI-1 and LRP1 [19]. TM5441, which is a derivative of TM5275, also induced intrinsic apoptosis in human fibrosarcoma and colorectal cancer cell lines in vitro [20]. However, although the anti-tumor effects of these compounds have been clearly demonstrated, their low hydrophilicity constitutes a serious weakness with regard to their development for clinical application. Accordingly, SK-216 has the advantage of being water soluble, and thus may be more useful for systemic administration in clinical practice. In addition, Masuda et al. reported that the systemic administration of SK-216 exerted an antitumor effect through its interaction with host PAI-1 [8]. Therefore, such a point should be examined in our mice model to reveal the mechanism of anti-tumor action of SK-216 comprehensively before clinical practice.

In conclusion, we identified that SK-216 may be a novel anti-metastasis agent for human osteosarcoma. Furthermore, the level of PAI-1 expression is correlated with malignancy in various cancers, such as breast [21], colorectal [22], rectal [23], gastric [24], renal cell [25], lung [26], cervical [27], head and neck [28], glioma [29], and ovarian [30] cancers. Therefore, suppressing PAI-1 expression may contribute to the improvement of prognosis in many common cancers, potentially indicating SK-216 as a novel broad-spectrum anti-cancer drug to prevent cancer progression in various human malignant tumors as well.

## 4. Materials and Methods

### 4.1. Reagents

A small compound PAI-1 inhibitor, SK-216 (Disodium [5-[[6-[5-(1,1-Dimethylethyl)-2-benzoxazolyl]-2-naphthalenyl]oxy]pentyl]propanedioate) was chemically synthesized and supplied by Shizuoka Coffein Co., Ltd. (Shizuoka, Japan). For in vivo experiments, SK-216 was dissolved in phosphate buffered saline at a concentration of 0.033 mg/mL, or in sterilized water and diluted in culture media for in vitro experiments.

### 4.2. Cell Culture and Transfection

The human osteosarcoma cell line 143B was obtained from the American Type Culture Collection (Manassas, VA, USA) and authenticated by JCRB (Japanese Collection of Research Bioresources) Cell Bank using short tandem repeat (STR) analysis. It was maintained in Dulbecco’s modified Eagle’s medium (DMEM; Nissui Pharmaceutical Co., Tokyo, Japan) containing 10% heat-inactivated fetal bovine serum at 37 °C in a humidified incubator with 5% CO_2_. The 143B cells were transfected with a complex of the pLuc-Neo plasmid DNA (143B-Luc) as described previously [3]. PAI-1 siRNA: 5′-AAGCACAACUCCCUUAAGGUCT-3′, or a random siRNA (Negative control siRNA#1, No. AM4636; Ambion, Austin, TX, USA) were each transfected into 143B cells at a concentration of 90 nM per 2.5 × 10^5^ cells in a 6-cm dish using DharmaFECT (GE Healthcare, Buckinghamshire, UK), according to the manufacturer’s instructions.

### 4.3. Cell Proliferation Assay

143B cells were plated at a density of 1.0 × 10^3^ cells/well in a 96-well plate. At 5 h after seeding, the media were changed to DMEM containing SK-216 (0, 25, or 50 μM) and proliferation was measured every 24 h using Cell counting Kit-8 (Dojindo, Kumamoto, Japan) according to manufacturer protocol.

### 4.4. Cell Migration Assay

143B cells were plated at a density of 5.0 × 10^5^ cells/well in 6-well plates and after 5 h, the media were changed to DMEM containing SK-216 (0, 25, or 50 μM). The next day, confluent cell monolayers were scratched using a sterile 1000 μL pipette tip using a CELL Scratcher^TM^ scratch guide (IWAKI, Tokyo, Japan). The wounds were captured immediately upon being scratched. After about 30 h, the wounds area was measured using software (BZ-analysis Application, Keyence, Osaka, Japan).

### 4.5. Cell Invasion Assay

The cell invasion assay was performed using the CytoSELECT 96-Well Cell Invasion Assay kit (Cell Biolabs, San Diego, CA, USA). 143B cells were seeded into 6-well plates at a density of 2.5 × 10^5^ cells/well. At 5 h after seeding, the media were changed to DMEM containing SK-216 (0, 25, or 50 μM). At 48 h after treatment, the cells were harvested and reseeded at a density of 2.5 × 10^5^ cells/well in a 96-well chambers following manufacturer protocol. After hematoxylin staining, cells that had invaded into Matrigel-coated pores were ascertained through coating pores with stained cells. Invasion indices were calculated as follows; pores with stained cells/total pores.

### 4.6. Western Blotting

143B cells were seeded into 6-well plates at a density of 2.5 × 10^5^ cells/well. After 5 h, the media were changed to DMEM containing SK-216 (0, 25, or 50 μM) and incubated another 48 h. SK-216 treated or siRNA-transfected cells were lysed on ice for 30 min with a lysis buffer (20 mM Tris-HCl pH 7.4, 150 mM NaCl, 0.1% sodium dodecyl sulphate, 1% sodium deoxycholate, 1% Triton, 1 mg Aprotinin, 1 mg Leupeptin). The cell lysates were centrifuged at 18,000× *g* for 5 min. The protein concentration was determined by means of the Bradford protein assay (Bio-Rad Laboratories, Carlsbad, CA, USA) using bovine serum albumin as the standard. Protein was resolved by electrophoresis through 12% polyacrylamide gels at 25 μg/lane, and electrotransferred to a polyvinylidene difluoride membrane (Millipore, Bedford, MA, USA). After blocking with 5% skim milk in PBS containing 1% Tween 20, the membranes were incubated over night at 4 °C with primary antibody. Following second antibody incubation, the membranes were rinsed and bound antibodies were detected using ECL prime western blotting detection reagent (GE Healthcare). The images were acquired using Image Quant LAS 4000 (Fuji Film, Tokyo, Japan). The primary antibodies that were used in this study were goat anti-human PAI-1 polyclonal antibody (1:400 dilution; ab31280; Abcam, Cambridge, UK) and mouse anti-β-actin (1:2000; AC-15; Sigma-Aldrich, St. Louis, MO, USA). The secondary antibodies used in this study were anti-goat IgG-horseradish peroxidase (HRP) (sc-2020; Santa Cruz Biotechnology, Dallas, TX, USA), and anti-mouse IgG-HRP (PM009-7; MBL, Nagoya, Japan).

### 4.7. Measurement of Secreted Matrix Metalloproteinase-13 (MMP-13) Protein

The level of MMP-13 protein secreted into the conditioned media of cultured 143B cells was determined using the SensoLyte 520 MMP-13 Assay Kit (Anaspec, San Jose, CA, USA). Briefly, the conditioned media were collected 72 h after SK-216 treatment. To measure total MMP-13 level, 1 mM *p*-aminophenylmercuric acetate was added into the collected media. The media were added to each microplate well, followed by the addition of the MMP-13 substrate and the 5-FAM/QXL520 FRET peptide. The fluorescence intensity representing MMP-13 expression was measured at 490/520 nm wavelengths.

### 4.8. Animal Model

All of the animal experiments were approved by the Institutional Animal Care and Use Committee of Tottori University (permit number: 13-Y-34). We anesthetized 5–6-week-old male athymic nude mice (CLEA Japan, Shizuoka, Japan) by exposure to 3% isoflurane on day 0 and subsequent days, as indicated. On day 0, to generate the experimental model, the anesthetized mice were injected with 3.0 × 10^6^ 143B-Luc cells into the knee of the right hind limb. Individual mice were injected with PBS or SK-216 solution intraperitoneally in a volume of 200 μL once every 3 days. The first injection was performed on day 1 post-inoculation of 143B-Luc cells. For in vivo imaging, the mice were injected with d-luciferin (150 mg/kg; Promega, Madison, WI, USA) by subcutaneous injection. After 15 min, photons from firefly luciferase were counted using the IVIS imaging system (Xenogen, Alameda, CA, USA) according to the manufacturer’s instructions. Data were analyzed using Living Image software (Xenogen). The development of subsequent lung metastases was monitored once every week in vivo by bioluminescent imaging for five weeks. At the end of the experiment on day 35, the primary tumors and lungs of each animal were resected at necropsy for histological and western blot analyses.

### 4.9. Histological Analysis of Tumors and Lung Metastases

All of the tumors and lungs resected from mice were fixed with 10% buffered formalin and embedded in paraffin. Thick sections of 3 μm were examined using histological analyses. The metastatic areas on hematoxylin and eosin stained lung sections were measured using software (BZ-analysis Application). Immunohistochemistry analyses were performed using tumor sections. Antigens were retrieved by autoclaving in 10 mM citrate buffer (pH 6.0) at 121 °C for 10 min. Endogenous peroxidase activity was blocked by immersing the slides in 0.6% hydrogen peroxide in methanol for 20 min. The primary antibody used in this study was a monoclonal antibody against human Ki-67 (Nichirei, Tokyo, Japan). Immunoreactions were visualized with diaminobenzidine and the sections were counterstained with hematoxylin.

### 4.10. Statistical Analyses

Statistical analyses were conducted using one-way analysis of variance (ANOVA) with Tukey-Kramer multiple comparison test for in vitro screening of cell invasion, migration, and MMP-13 secretion. A Student’s *t*-test was used to evaluate PAI-1 expression and Ki-67 expression analysis in vivo. A Welch’s *t*-test was used to evaluate the lung metastasis area in vivo assay. The Mann-Whitney U test was used for in vitro screening of cell proliferation. Differences in lung metastatic frequency were evaluated with a Chi-squared test. *p* < 0.05 was considered significant.

## Figures and Tables

**Table 1:** Lung metastasis positive mice/total mice.

	IVIS	Macroscopy
Control	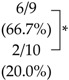	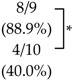
SK-216		

The positive cases for lung metastasis were significantly reduced in the SK-216-treated group. * *p* < 0.05. IVIS: in vivo imaging system.

## Supplementary Materials

Supplementary materials can be found at http://www.mdpi.com/1422-0067/19/3/736/s1.
