# Supplementary material for: SK-216, a Novel Inhibitor of Plasminogen Activator Inhibitor-1, Suppresses Lung Metastasis of Human Osteosarcoma
Source: Int J Mol Sci. 2018 Mar 5;19(3):736. doi: 10.3390/ijms19030736 (PMC5877597; doi:10.3390/ijms19030736)
Supplement: Supplementary file 1 [file ijms-19-00736-s001.pdf]

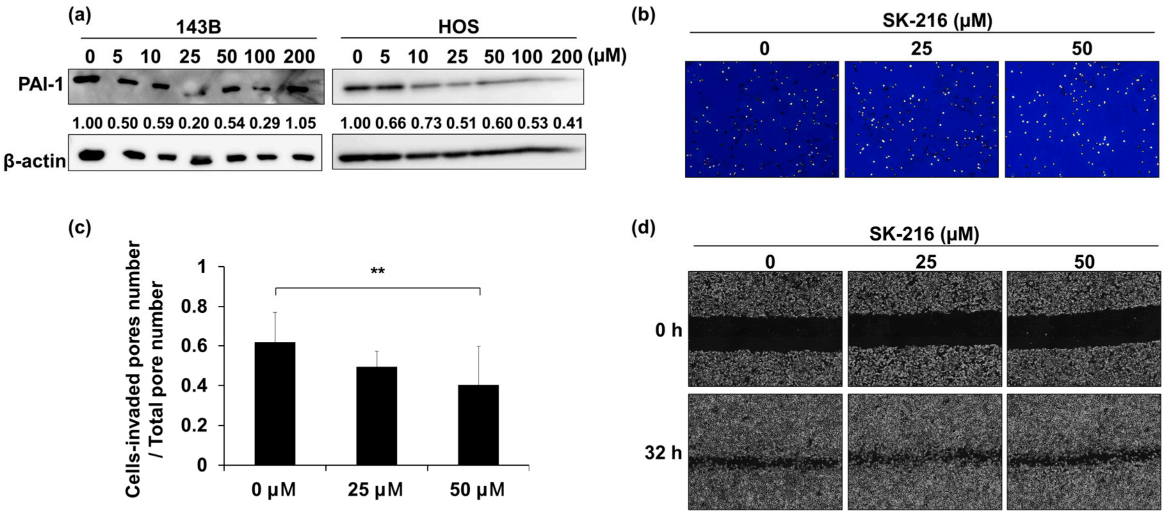

Supplementary Figure S1. (a) Western blot analyses of PAI-1 expression in SK-216-treated osteosarcoma cell lines. PAI-1 expression was quantified using Image Studio Lite (LI-COR) and normalized to β-actin. Expression is shown relative to that in non-treated cells (0 μM). (b) The representative images of invasion assay of SK-216 treated 143B cells were shown. The hematoxylin-stained cells in pores ascertained through coating pores. (c) Matrigel assay of the invasion of SK-216-treated HOS cells. The ratio of the number of pores containing invading cells to the total number of all pores is shown. Bar graphs show means ± SD **P<0.01. (d) The representative images of scratch assay of SK-216 treated 143B cells were shown.

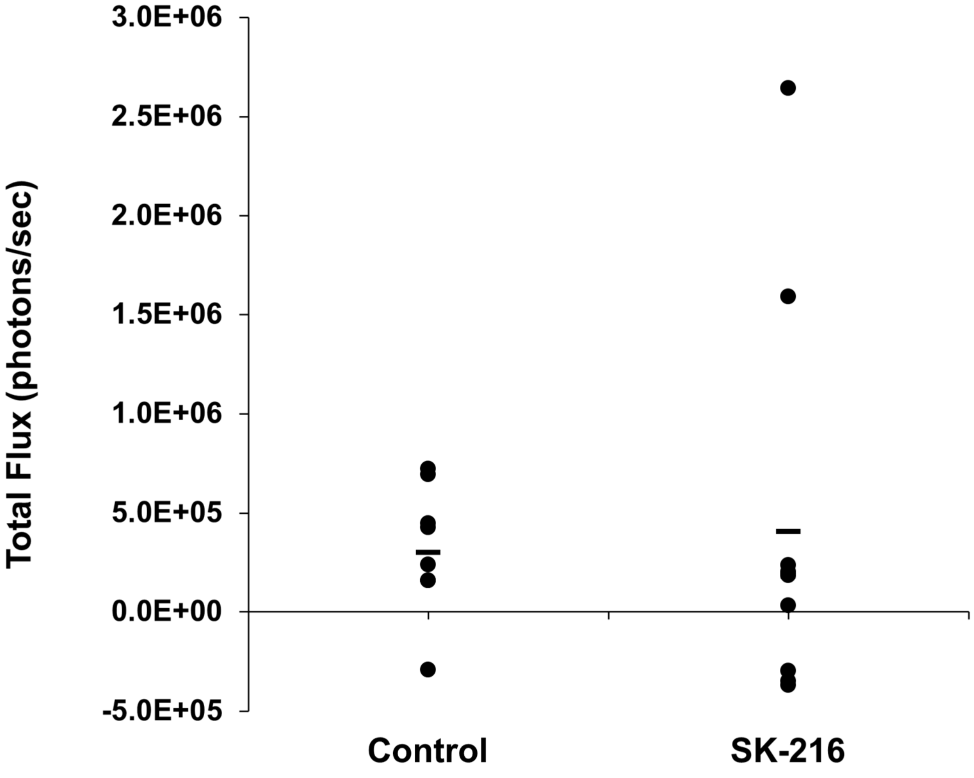

Supplementary Figure S2. Total Flux (photons/sec) in the lungs were measured at 5 weeks after cell inoculaton. The black bar indicates the mean value.
